# Origin and Evolution of Nitrogen Fixation Genes on Symbiosis Islands and Plasmid in *Bradyrhizobium*

**DOI:** 10.1264/jsme2.ME15159

**Published:** 2016-07-12

**Authors:** Takashi Okubo, Pongdet Piromyou, Panlada Tittabutr, Neung Teaumroong, Kiwamu Minamisawa

**Affiliations:** 1 Environmental Biofunction Division, National Institute for Agro-Environmental Sciences3–1–3 Kannondai, Tsukuba, Ibaraki 305–8604Japan; 2 Graduate School of Life Sciences, Tohoku University2–1–1 Katahira, Aoba-ku, Sendai, Miyagi 980–8577Japan; 3 Institute of Agricultural Technology, School of Biotechnology, Suranaree University of TechnologyNakhon Ratchasima 30000Thailand

**Keywords:** nitrogen fixation, *Bradyrhizobium*, nodule symbiosis, evolution, guanine and cytosine content

## Abstract

The nitrogen fixation (*nif*) genes of nodule-forming *Bradyrhizobium* strains are generally located on symbiosis islands or symbiosis plasmids, suggesting that these genes have been transferred laterally. The *nif* genes of rhizobial and non-rhizobial *Bradyrhizobium* strains were compared in order to infer the evolutionary histories of *nif* genes. Based on all codon positions, the phylogenetic tree of concatenated *nifD* and *nifK* sequences showed that *nifDK* on symbiosis islands formed a different clade from *nifDK* on non-symbiotic loci (located outside of symbiosis islands and plasmids) with elongated branches; however, these genes were located in close proximity, when only the 1st and 2nd codon positions were analyzed. The guanine (G) and cytosine (C) content of the 3rd codon position of *nifDK* on symbiosis islands was lower than that on non-symbiotic loci. These results suggest that *nif* genes on symbiosis islands were derived from the non-symbiotic loci of *Bradyrhizobium* or closely related strains and have evolved toward a lower GC content with a higher substitution rate than the ancestral state. Meanwhile, *nifDK* on symbiosis plasmids clustered with *nifDK* on non-symbiotic loci in the tree representing all codon positions, and the GC content of symbiotic and non-symbiotic loci were similar. These results suggest that *nif* genes on symbiosis plasmids were derived from the non-symbiotic loci of *Bradyrhizobium* and have evolved with a similar evolutionary pattern and rate as the ancestral state.

Rhizobial bacteria establish nodule symbiosis with legumes. Inside nodules, rhizobia fix atmospheric dinitrogen and provide fixed nitrogen to the plant. It is generally hypothesized that rhizobial genes related to nodulation (*nod*) and symbiotic nitrogen fixation (*nif*) were acquired by lateral gene transfer in *Bradyrhizobium* because these genes are clustered in a large genomic island known as a symbiosis island, which is characterized by a lower GC content than the rest of the genome (10, 11).

To date, the complete genome sequences of four *nod*-lacking *Bradyrhizobium* strains have been elucidated: *Bradyrhizobium* spp. S23321 (19), S58 (20), ORS278, and BTAi1 (5). These strains do not possess symbiosis islands. However, *nif* gene clusters (*nif*–clusters) are present in their chromosomes, which were likely to have been vertically transferred from their ancestors (19). The organization of *nif*–clusters is very similar among these strains (17, 19, 20), but differ from those on symbiosis islands of other strains such as *B. diazoefficiens* USDA110 (19). Hereafter, we designate the location of genes outside of the symbiotic region (symbiosis island or symbiosis plasmid) as “non-symbiotic loci.” A phylogenetic analysis of *nifH* (encoding nitrogenase iron protein) showed that *nifH* on non-symbiotic loci formed a different clade from those on symbiosis islands (19). These results suggest that the evolutionary history of *nif* genes is different between those found on non-symbiotic loci and on symbiosis islands.

A previous study showed that symbiotic genes (*e.g.*, *nod* and *nif*) in *Bradyrhizobium* sp. DOA9 were located on a large-sized plasmid (approximately 736 kb), known as the symbiosis plasmid, providing the first evidence for symbiotic genes being located on a plasmid in *Bradyrhizobium* strains (17, 23). Strain DOA9 possesses two *nif*–clusters in its genome. One is located on the symbiosis plasmid (symbiotic loci) and the other is located on the chromosome (non-symbiotic loci) (17). The nucleotide sequences of *nifH* on the plasmid and chromosome share 92% sequence identity (17). A phylogenetic analysis revealed that *nifH* in the plasmid and chromosome of strain DOA9 clustered together with *nifH* genes located in the non-symbiotic loci of other *Bradyrhizobium* strains: S23321, S58, ORS278, and BTAi1 (17). These results indicate that the evolutionary histories of *nif* genes on symbiosis islands and plasmids are distinct.

In the present study, the *nif* genes of rhizobial and non-rhizobial *Bradyrhizobium* strains were compared to infer the evolutionary histories of *nif* genes using various *Bradyrhizobium* strains including newly genome-sequenced strains: *Bradyrhizobium* sp. DOA1 (a rhizobial strain possessing divergent nodulation genes [15]) and *Bradyrhizobium* sp. MAFF107635 (a synonym of AT1; a non-rhizobial strain isolated from the storage roots of sweet potato [24]).

## Materials and Methods

### Genome sequencing and analyses of strains DOA1 and MAFF107635

Strains DOA1 and MAFF107635 were cultured to the stationary phase at 30°C in HM salt medium (3) containing 0.1% arabinose and 0.025% yeast extract. The cells were harvested by centrifugation, and total DNA was prepared as previously described (14). The 8-kb paired-end 454 libraries of strains DOA1 and MAFF107635 were then constructed from total DNA by Takara Bio (Shiga, Japan). Genome sequencing was performed using a 454 GS FLX+ sequencer (Roche Diagnostics; Tokyo, Japan). The generated sequences were assembled using Roche GS De Novo Assembler version 2.5p1 (Roche Diagnostics). Two potentially contaminated regions from primer or adaptor sequences used in 454 sequencing were detected in the strain DOA1 genome at positions 1625647–1625666 and 6325190–6325209, both of which were located at the edge of contigs. The nucleotides of these regions were changed into Ns. Predictions of gene regions and annotations were performed using NCBI Prokaryotic Genome Automatic Annotation Pipeline (PGAP) (1). A circular genome map showing the GC skew and GC content was created using arcWithColor version 1.3.8 (http://www.ige.tohoku.ac.jp/joho/gmProject/gmdownload.html). GC content and skew were calculated with sliding windows along the genome. The window size was set to 20,000 bases and the step size to 500 bases. The chromosome sequences of strains DOA1 and MAFF107635 containing runs of Ns that represent gaps were used as inputs. All predicted gap lengths were less than 5 kb. Organizations of *nif*–clusters were compared using GenomeMatcher version 1.861 (16).

### Tested strains for phylogenetic and molecular evolutionary analyses

The tested strains for phylogenetic and molecular evolutionary analyses are listed in Table 1. In addition to the newly genome-sequenced strains MAFF107635 and DOA1, we used *Bradyrhizobium* strains whose complete or high-quality draft (less than 10 scaffolds) genome sequences have been deposited in the NCBI genome database (last accessed April 2015). Since complete or high-quality draft genomes were limited to a narrow phylogenetic range of *Bradyrhizobium* species (Table 1), *B. yuanmingense* CCBAU 35157 and *B. liaoningense* CCBAU83689 were added to the tested strains even though their genomes are comprised of more than 10 scaffolds. The genomes of other *Bradyrhizobium* species were unavailable in the NCBI genome database.

A symbiosis island is defined as a DNA segment containing a cluster of symbiotic genes (*nif* and/or *nod*) with a lower GC content than the entire genome (Table 1) (10). The genomic locations of *nif* genes are elucidated based on these criteria.

### Phylogenetic analysis of the 16S rRNA gene and concatenated housekeeping genes

16S rRNA gene sequences were aligned using the CLUSTAL W program in MEGA 6.06 (22). The phylogenetic tree was constructed using nucleotide sequences via the maximum likelihood method with 1,000 bootstrap replicates in MEGA 6.06. The model with the lowest Bayesian Information Criterion (BIC) score by the “Find Best DNA/Protein Models” procedure of MEGA 6.06 was applied. The same analyses were conducted using the concatenated nucleotide sequences of four housekeeping genes (*rpoB*, *recA*, *gyrB*, and *atpD*).

### Phylogenic and molecular evolutionary analysis of concatenated *nifDK* sequences

The concatenated sequences of *nifD* (encoding the alpha subunit of the nitrogenase molybdenum-iron protein) and *nifK* (encoding the beta subunit of the nitrogenase molybdenum-iron protein) were analyzed in order to examine the phylogenetic relationships of *nif* genes in the tested strains. *nifH* was not used in the phylogenetic analysis because the tested strains, S23321, S58, ORS278, and BTAi1, possess two copies of *nifH* in the *nif*–cluster, which may affect the substitution rates of *nifH* sequences and lead to the incorrect estimation of phylogenetic relationships. The phylogenetic tree was constructed using nucleotide sequences by the maximum likelihood method as described above. The same analyses were performed using the 1st + 2nd codon positions. *Rhodopseudomonas palustris CGA009* was used as an outgroup because it is phylogenetically close to *Bradyrhizobium* and possesses a *nif*-cluster, which may be descended from a common ancestor of the *nif*-cluster loci of *Bradyrhizobium* strains on non-symbiotic loci. The neighbor-joining method was also applied to confirm the tree topology. Evolutionary distances were computed using the Tamura 3-parameter method. Differences in the composition bias among sequences were considered in evolutionary comparisons.

Disparity index tests of substitution pattern homogeneity were performed for each pair of concatenated nucleotide sequences of *nifDK* using MEGA 6.06 (22). A Monte Carlo test (1,000 replicates) was used to estimate *P*-values. *P*-values smaller than 0.01 were considered significant. The nucleotide compositions of each of the concatenated *nifDK* sequences were computed using MEGA 6.06. The same analyses were performed for the 1st + 2nd codon position. The molecular clock test was performed for tree topologies representing all and the 1st + 2nd codon positions by comparing the maximum likelihood value with and without the molecular clock constraints using MEGA 6.06. Codon-based tests of positive selection were performed using MEGA 6.06 in order to compare the number of synonymous and non-synonymous nucleotide substitutions.

### Accession numbers

The BioProject for genome sequencing has been deposited at DDBJ/EMBL/GenBank under the accession numbers PRJNA271717 for strain DOA1 and PRJNA271718 for strain MAFF107635. The BioSample has been deposited at DDBJ/EMBL/GenBank under the accession number SAMN03276511 for strain DOA1 and SAMN03276517 for strain MAFF107635. The genome sequences have been deposited at DDBJ/EMBL/GenBank under the accession number JXJM01000000 for strain DOA1 and JXDL01000000 for strain MAFF107635.

## Results and discussion

### Phylogenetic diversity of tested strains

**Fig. 1:**
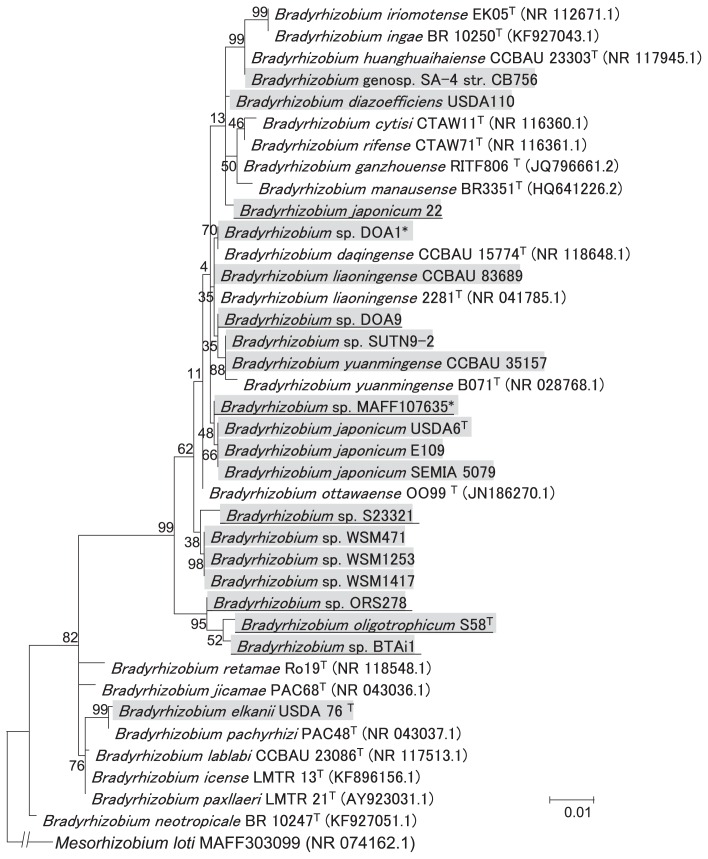
Phylogenetic tree by the maximum likelihood method for the 16S rRNA gene of tested and type strains in *Bradyrhizobium* species. Numbers at the nodes are the percentages of 1,000 bootstrap replications supporting the partition. The tested strains are shaded in gray. The tested strains possessing *nifDK* on non-symbiotic loci are underlined. The numbers in parentheses show sequence ID in public databases. *Mesorhizobium loti* MAFF303099 (NR_074162.1) was used as an outgroup. The newly genome-sequenced strains DOA1 and MAFF107635 are marked with asterisks.

A phylogenetic tree of the 16S rRNA gene sequences was constructed using the tested strains and type strains of *Bradyrhizobium* species (Fig. 1). The tested strains (grey shadow in Fig. 1) appear to cover a clade including *B. diazoefficiens*, *B. japonicum*, *B. yuanmingense*, and *B. oligotrophicum* (Fig. 1). Thus, our data set provides some insights into the evolution of *nif* genes in *Bradyrhizobium*, as described below.

### Genome sequencing of strains DOA1 and MAFF107635

**Fig. 2:**
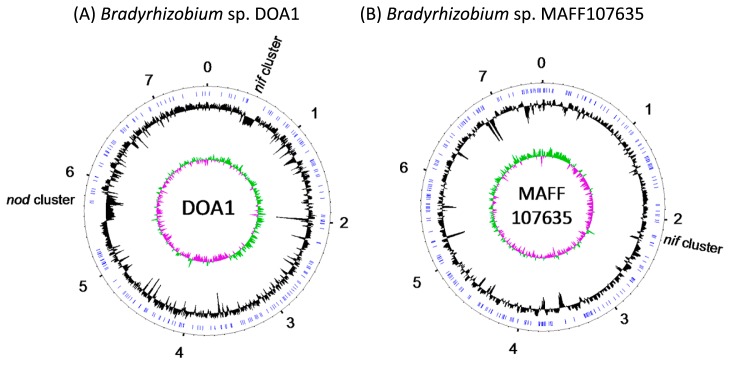
Circular view of whole-genome sequences of *Bradyrhizobium* sp. DOA1 (A) and MAFF107635 (B) chromosomes. The outermost circle represents genome positions in megabases. The second-outermost circle illustrates the positions of gap regions (blue). The innermost and second-innermost circles illustrate the GC (guanine-cytosine) skew (green and purple) and GC content (black), respectively.

The draft genome sequences of strains DOA1 and MAFF107635 were determined using 8-kb paired-end 454 pyrosequences. The read assemblies of strain DOA1 produced one circular scaffold consisting of 201 contigs. Two large low-GC regions were found in the 450–550-kb and 5,600–5,800-kb regions and contained the *nif*–cluster and *nod* genes, respectively (Fig. 2A). These characteristics strongly suggest that these regions are symbiosis islands.

The read assemblies of strain MAFF107635 produced one circular scaffold consisting of 260 contigs. The *nif* genes were located in the 2,193–2,226-kb region (Fig. 2B). The GC content of this region was similar to that of the whole genome. No symbiosis island or plasmid was found in the genome of strain MAFF107635 (Fig. 2B).

### Phylogenic analysis of 16S rRNA and housekeeping gene sequences

**Fig. 3:**
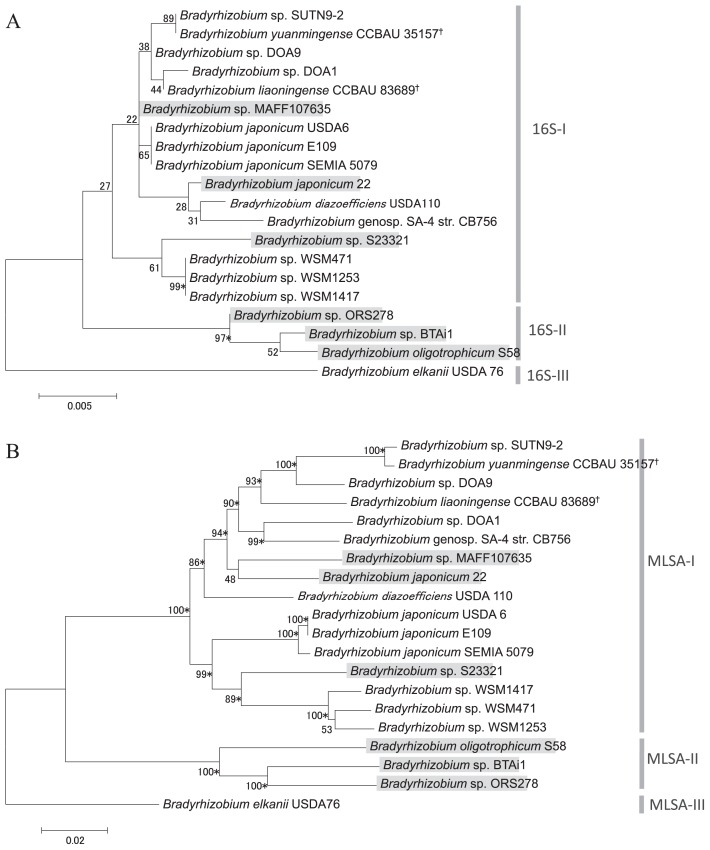
Phylogenetic trees by the maximum likelihood method for the 16S rRNA gene (A) and for concatenated sequences of four housekeeping genes (*rpoB*, *recA*, *gyrB*, and *atpD*) (B). Numbers at the nodes are the percentages of 1,000 bootstrap replications supporting the partition. Strains possessing *nifDK* on non-symbiotic loci are shaded in gray. Asterisks following the bootstrap values show that the branches are supported by the neighbor-joining method with ≥70% bootstrap values. Strains whose genomic locations of *nifDK* are unknown are marked with †.

The phylogenetic tree of 16S rRNA gene sequences separated the tested *Bradyrhizobium* strains into three groups (Fig. 3A). Group 16S-I was comprised of three non-rhizobial strains including the newly sequenced strain MAFF107635 and 13 rhizobial strains including the newly sequenced strain DOA1. Group 16S-II was comprised of three strains, all of which have the ability to establish nodule symbiosis on some species of *Aeschynomene* plants (*e.g.*, *Aeschynomene indica* and *A. evenia*) without nodulation factor signals (5, 18, 20); none of the strains in this group possessed symbiosis islands or plasmids. The rhizobial strain USDA76 formed a separate group from the other strains. The tree of 16S rRNA genes suggested that *nod* gene-lacking and-possessing strains were not clearly separated because the *nod* gene-lacking strains MAFF107635, S23321, and 22 were sporadically distributed in group 16S-I along with *nod* gene-possessing strains. However, the phylogenetic tree of 16S rRNA genes showed less than 70% bootstrap support for all branches in group 16S-I. In order to achieve better phylogenetic resolution, we applied a multilocus sequence analysis (MLSA) approach (4). The tree of the concatenated sequences of four housekeeping genes (*rpoB*, *recA*, *gyrB*, and *atpD*) showed 70% bootstrap support for most branches (Fig. 3B). The MLSA tree separated the tested strains into three groups consisting of the same members as the 16S rRNA gene tree. The non-rhizobial strains MAFF107635, S23321, and 22 were clustered together with *nod* gene-possessing strains in the group MLSA-I. These results show that *nod* gene-lacking and-possessing strains are closely related based on the sequences of the marker genes used for the phylogenetic analysis.

### Phylogenetic analyses of concatenated *nifDK* sequences

**Fig. 4:**
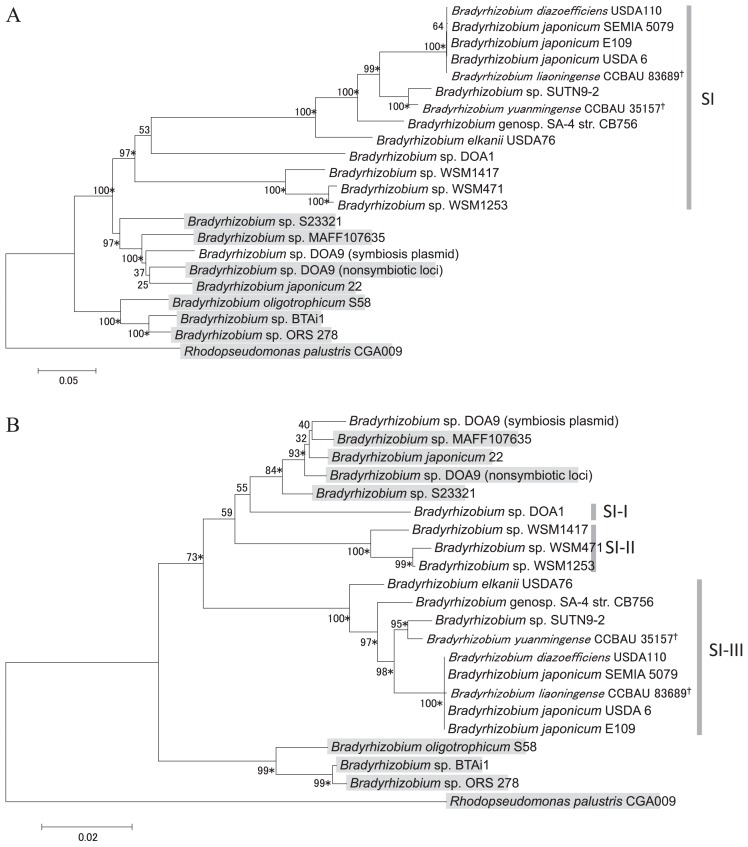
Phylogenetic trees by the maximum likelihood method for concatenated *nifDK* sequences representing all codon positions (A) and representing only the 1st and 2nd codon positions (B). Numbers at the nodes are the percentages of 1,000 bootstrap replications supporting the partition. *nifDK* on non-symbiotic loci are shaded in gray. Asterisks following the bootstrap values show that the branches are supported by the neighbor-joining method with ≥70% bootstrap values. Strains whose genomic locations of *nifDK* are unknown are marked with †.

In order to infer the phylogenetic relationships of *nif* genes in the tested strains, we analyzed the concatenated sequences of *nifD* and *nifK*. *nifDK* on the symbiosis plasmid of strain DOA9 was clustered with those on the non-symbiotic loci of strain DOA9 and other strains (Fig. 4A), suggesting that the *nif* genes found on the symbiosis plasmid of strain DOA9 were derived from non-symbiotic loci of *Bradyrhizobium*.

**Fig. 5:**
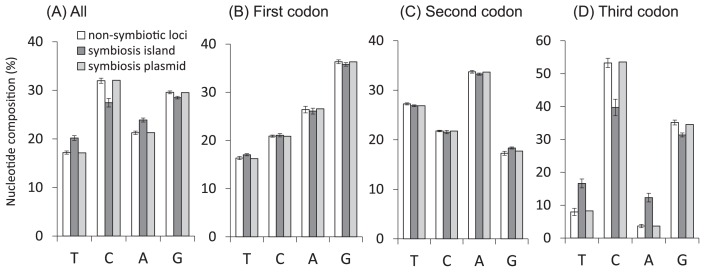
The average nucleotide composition for concatenated *nifDK* sequences located on non-symbiotic loci, symbiosis islands, and symbiosis plasmids. The average base compositions of all positions (A), the first codon position (B), second codon position (C), and third codon position (D) are shown in each panel. The error bars represent standard deviations. *n*=7 for non-symbiotic loci, *n*=11 for symbiosis islands, *n*=1 for symbiosis plasmids.

*nifDK* genes on symbiosis islands formed a monophyletic group with 97% bootstrap support (Fig. 4A), whereas the same strains were more distinctly distributed according to 16S rRNA (Fig. 3A) and concatenated housekeeping gene (Fig. 3B) trees. The branches of *nifDK* on symbiosis islands were elongated (Fig. 4A). These results indicate the different evolutionary histories of *nifDK* on symbiosis islands and non-symbiotic loci. The nucleotide composition analysis of *nifDK* showed that *nifDK* on symbiosis islands had a lower GC content than on non-symbiotic loci (Fig. 5A). In addition, the molecular clock test rejected the equal evolutionary rate model throughout the tree (*P*<0.001). These results suggest two possible scenarios for *nifDK* on symbiosis islands. One scenario is that *nifDK* on symbiosis islands was inherited from a different ancestor from non-symbiotic loci with a low GC content. The other scenario is that *nifDK* on symbiosis islands was inherited from the same ancestor with non-symbiotic loci; however, it has since evolved toward a lower GC content with a higher substitution rate than *nifDK* on non-symbiotic loci.

### Molecular evolutionary analyses of concatenated *nifDK* sequences

In order to examine whether the pattern of nucleotide substitution is constant in *nifDK* on symbiosis islands and non-symbiotic loci, the disparity index tests of substitution pattern homogeneity (12) were performed for each pair of *nifDK* sequences (Table 2). In the disparity index tests using all (1st+2nd+3rd) codon positions, the null hypothesis that sequences have evolved with the same pattern of substitutions was rejected for all pairs of *nifDK* sequences in the comparison of symbiosis islands and non-symbiotic loci, suggesting that *nifDK* on symbiosis islands have evolved via markedly different evolutionary processes from those on non-symbiotic loci. In contrast, the acceptance rate of the null hypothesis was 100% in the comparison of symbiosis plasmids and non-symbiotic loci, suggesting that *nifDK* on non-symbiotic loci and symbiosis plasmids have evolved via the same or similar evolutionary processes. The acceptance rate of the null hypothesis was 87% among symbiosis islands, suggesting that the substitution pattern may not be constant among *nifDK* on symbiosis islands. The acceptance rate of the null hypothesis for all pairs of tested strains was 44%, which increased to 99% when only the 1st or 2nd codon position was analyzed, suggesting that *nifDK* on symbiosis islands and non-symbiotic loci have evolved with the same or similar pattern of substitutions in the 1st and 2nd codon positions only. A nucleotide composition analysis of *nifDK* showed that *nifDK* on symbiosis islands had a lower GC content than those in non-symbiotic loci (Fig. 5A). This difference was particularly notable at the 3rd codon position (Fig. 5B, C, and D). The results of disparity index tests and nucleotide composition analyses suggested that *nifDK* in symbiosis islands and non-symbiotic loci have evolved via similar evolutionary processes at the 1st and 2nd codon positions.

We next analyzed the concatenated sequences of *nifDK* using only the 1st and 2nd codon positions to alleviate the effects of heterogeneous sequence evolution among the tested strains. The tree representing the 1st and 2nd codon positions (Fig. 4B) showed a different topology from the tree representing all positions (Fig. 4A). In the 1st and 2nd codon positions, *nifDK* on symbiosis islands were clustered into three groups and formed a polyphyly (Fig. 4B). The branch length elongation of *nifDK* on symbiosis islands was alleviated (Fig. 4A and B), suggesting that the long branches of *nifDK* on symbiosis islands in the tree representing all codon positions mainly resulted from heterogeneous sequence evolution in the 3rd codon position. *nifDK* on symbiosis islands was located close to *nifDK* on non-symbiotic loci, suggesting that *nifDK* on symbiosis islands was derived from the non-symbiotic loci of *Bradyrhizobium* or closely related strains. It is also noteworthy that the branches of *nifDK* on symbiosis islands were still slightly elongated, suggesting that heterogeneous sequence evolution may occur not only in the 3rd codon position, but also in the 1st and 2nd codon positions, which is supported by the molecular clock test showing that the evolutionary rate is not equal throughout the tree in the 1st and 2nd codon positions (*P*<0.001). Codon-based tests of positive selection showed that the number of synonymous nucleotide substitutions exceeds the number of non-synonymous nucleotide substitutions in all sequence pairs (*P*>0.05), which will lead to a higher substitution rate in the 3rd codon position containing a large number of synonymous sites. Although *nifDK* on symbiosis islands formed a polyphyly in the phylogenetic tree representing the 1st and 2nd codon positions (Fig. 4B), this does not necessary indicate a polyphyletic origin of *nif*–clusters on symbiosis islands because we cannot exclude the possibility that heterogeneous evolution occurred among *nifDK* genes located on symbiosis islands, which may also lead to a polyphyletic relationship. The hypothesis of the monophyletic origin of *nif*–clusters in symbiosis islands is supported by previous studies showing highly congruent trees for *nifH* and *nod* genes (*nodYK*, *nodA*, and *nodZ*) (13) and high linkage disequilibrium values for *nifD*, *nifH*, and *nodC* in symbiosis islands (21).

The disparity index (Table 2) and nucleotide composition (Fig. 5) analyses indicated that *nifDK* on symbiosis plasmids are more closely related to *nifDK* on non-symbiotic loci than to those on symbiosis islands, suggesting different evolutionary histories for *nifDK* on symbiosis islands and plasmids. However, this hypothesis needs to be carefully evaluated using more strains because only one of the strains tested herein possessed a symbiosis plasmid (Table 1).

### Origin of *nif* genes on the symbiosis island

**Fig. 6:**
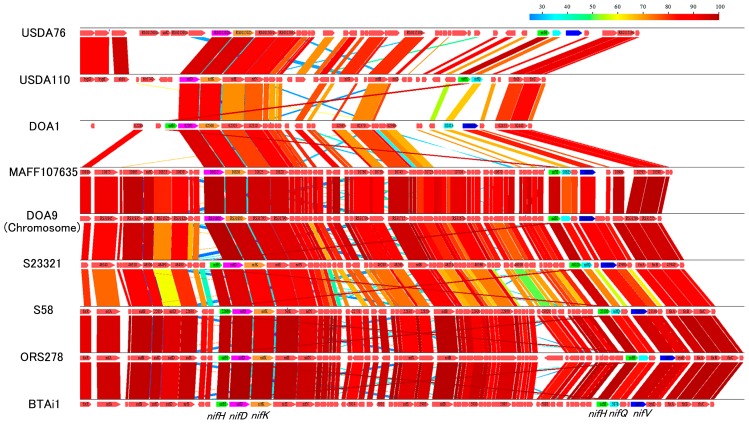
Blastp comparisons of *nif*–clusters. The color scale indicates the percent similarity. The arrows show each gene. *nifH* genes are shown in green, *nifD* genes are shown in magenta, *nifK* genes are shown in orange, *nifQ* genes are shown in cyan, *nifV* genes are shown in blue.

In order to test the plausibility that the origin of the *nif*–cluster in symbiosis islands is the non-symbiotic loci of *Bradyrhizobium*, we compared the *nif*–cluster of strains DOA1, USDA110, and USDA76 in the symbiosis island with those in the non-symbiotic loci of the other *Bradyrhizobium* strains MAFF107635, DOA9, S23321, S58, ORS278, and BTAi1. The organization of *nif*–clusters in symbiosis islands was very similar to those on non-symbiotic loci in other strains (Fig. 6), which supports the hypothesis of an origin from the non-symbiotic loci of *Bradyrhizobium*. In strain DOA1, *nifH* was located in an operon with *nifDK*, whereas in strains MAFF107635 and DOA9, *nifH* was located in an operon with *nifQ* (Fig. 6). In strains S23321, S58, ORS278, and BTAi1, *nifH* was located in operons with *nifDK* and *nifQ* (Fig. 6). These results suggest that the *nif*–cluster in the symbiosis island of strain DOA1 was laterally transferred from a strain possessing only the *nifHDK* operon. However, none of the tested strains only possess the *nifHDK* operon in non-symbiotic loci. These results suggest two possibilities: a rearrangement of gene organization occurred in the *nif*–cluster of strain DOA1 or we were unable to identify the strains possessing only the *nifHDK* operon because of the limited number of strains tested. Strains USDA110 and USDA76 only possess the *nifHQ* operon (Fig. 6). This feature was observed in the *nif*–clusters of strains MAFF107635 and DOA9 on non-symbiotic loci. The *nif*–cluster of strains DOA1 and USDA76 possesses *nifV*, which encodes homocitrate synthase (Fig. 6). Homocitrate is a component of the iron-molybdenum cofactor of nitrogenase (9). Most rhizobial strains do not possess *nifV*, which is complemented by homocitrate provided from the host plant during nodule symbiosis (7). The presence of *nifV* in strains DOA1 and USDA76 partially supports the hypothesis that the *nif*–cluster on symbiosis islands was derived from the non-symbiotic loci of *Bradyrhizobium* or closely related strains. However, we cannot absolutely rule out the possibility that *nif* genes on symbiosis islands and plasmids serve as the donors of *nif* genes on non-symbiotic loci. This possibility needs to be carefully examined by studying more *Bradyrhizobium* strains that possess *nif* genes at non-symbiotic loci.

## Conclusion

The findings of previous studies suggest that *nif* genes in symbiosis islands were acquired by lateral gene transfer because these genes are located in a symbiosis island, which is characterized by a lower GC content than the rest of the genome (10, 11). In the present study, we inferred the origin and evolutionary process of *nif* genes in *Bradyrhizobium* strains. Our analyses indicate that *nif* genes on symbiosis islands and plasmids were derived from the non-symbiotic loci of *Bradyrhizobium* or closely related strains. Following gene transfer, *nif* genes on symbiosis islands evolved faster than those in the ancestral state toward a lower GC content; however, we cannot absolutely rule out the possibility that *nif* genes on symbiosis islands were transferred from bacteria with a low genomic GC content, closely related to *Bradyrhizobium*. In contrast, no apparent substitution bias was observed in the *nif* genes on symbiosis plasmids. Previous studies (2, 6, 8) analyzed *nif* genes across diverse taxonomic groups of α- and β-proteobacteria, and reported that rhizobial and non-rhizobial nitrogen-fixing strains were clustered in the same clade in phylogenetic trees. However, the *nif* genes of nodulation bacteria are closely related to those of non-rhizobial strains of the same or taxonomically-related groups. These results suggest that the translocation or lateral transmission of *nif* genes from non-symbiotic loci to symbiosis islands or plasmids occurred not only in *Bradyrhizobium*, but also in other rhizobial groups. Due to the limited number of complete or high-quality draft *Bradyrhizobium* genomes, our data set does not cover all *Bradyrhizobium* species. Further studies using diverse strains and genes will contribute to developing a greater understanding of the origin and evolution of legume–rhizobia symbiosis.

## Figures and Tables

**Table 1:** Genomic locations of nitrogen fixation gene clusters in strains tested in the present study.

Strain	Type of symbiosis region	Location of the *nif*–cluster	*nod* gene	Accession number
*Bradyrhizobium* sp. DOA1	symbiosis island	symbiosis island	+	JXJM01000000
*Bradyrhizobium diazoefficiens* USDA110	symbiosis island	symbiosis island	+	BA000040
*Bradyrhizobium japonicum* USDA6	symbiosis island	symbiosis island	+	AP012206
*Bradyrhizobium japonicum* E109	symbiosis island	symbiosis island	+	CP010313
*Bradyrhizobium japonicum* SEMIA 5079	symbiosis island	symbiosis island	+	CP007569
*Bradyrhizobium elkanii* USDA76	symbiosis island	symbiosis island	+	KB900701, KB900702
*Bradyrhizobium* sp. WSM471	symbiosis island	symbiosis island	+	CM001442
*Bradyrhizobium* sp. WSM1253	symbiosis island	symbiosis island	+	JH600072, JH600073
*Bradyrhizobium* genosp. SA-4 str. CB756	symbiosis island	symbiosis island	+	AXBC00000000
*Bradyrhizobium* sp. WSM1417	symbiosis island	symbiosis island	+	AZXU00000000
*Bradyrhizobium* sp. SUTN9-2	symbiosis island	symbiosis island	+	LAXE00000000
*Bradyrhizobium liaoningense* CCBAU 83689	unknown^a^	unknown^a^	+	AJQD00000000
*Bradyrhizobium yuanmingense* CCBAU 35157	unknown^a^	unknown^a^	+	AJQL00000000
*Bradyrhizobium* sp. DOA9	symbiosis plasmid	non-symbiotic loci symbiosis plasmid	+	DF820425, DF820426
*Bradyrhizobium* sp. MAFF107635	—	non-symbiotic loci	−	JXDL01000000
*Bradyrhizobium* sp. S23321	—	non-symbiotic loci	−	AP012279
*Bradyrhizobium japonicum* 22	—	non-symbiotic loci	−	AXVG00000000
*Bradyrhizobium oligotrophicum* S58	—	non-symbiotic loci	−	AP012603
*Bradyrhizobium* sp. BTAi1	—	non-symbiotic loci	−	CP000494, CP000495
*Bradyrhizobium* sp. ORS278	—	non-symbiotic loci	−	CU234118

a Information was not obtained because the *nif*–cluster was located on a small contig.

**Table 2:** Number of concatenated *nifDK* sequence pairs for which the null hypothesis was accepted or rejected that sequences have evolved with the same pattern of substitution, as judged from a disparity index test

Comparison pair	All codon positions (1st+2nd+3rd)			1st and 2nd codon position		
	
	Number of pairs		Acceptance rate (%)	Number of pairs		Acceptance rate (%)
	
	Accept	Reject		Accept	Reject	
Symbiosis island vs. Non-symbiotic loci	0	77	0	77	0	100
Symbiosis plasmid vs. Non-symbiotic loci	7	0	100	7	0	100
Symbiosis island vs. Symbiosis island	48	7	87	55	0	100
Non-symbiotic loci vs. Non-symbiotic loci	20	1	95	20	1	95
Symbiosis plasmid vs. Symbiosis island	0	11	0	11	0	100

Total	75	96	44	170	1	99

*P*-values less than 0.01 were considered significant.

## Abbreviations

GC: guanine and cytosine
*nif* genes: nitrogen fixation gene
*nod* genes: nodulation genes
MLSA: multilocus sequence analysis
